# Adaptive variability in the duration of critical windows of plasticity: Implications for the programming of obesity

**DOI:** 10.1093/emph/eou019

**Published:** 2014-08-05

**Authors:** Jonathan C. K. Wells

**Affiliations:** Childhood Nutrition Research Centre, UCL Institute of Child Health, 30 Guilford Street, London WC1N 1EH, UK

**Keywords:** developmental plasticity, adaptation, critical window, parent–offspring conflict, growth, obesity

## Abstract

Developmental plasticity underlies widespread associations between early-life exposures and many components of adult phenotype, including the risk of chronic diseases. Humans take almost two decades to reach reproductive maturity, and yet the ‘critical windows’ of physiological sensitivity that confer developmental plasticity tend to close during fetal life or infancy. While several explanations for lengthy human maturation have been offered, the brevity of physiological plasticity has received less attention. I argue that offspring plasticity is only viable within the niche of maternal care, and that as this protection is withdrawn, the offspring is obliged to canalize many developmental traits in order to minimize environmental disruptions. The schedule of maternal care may therefore shape the duration of critical windows, and since the duration of this care is subject to parent*–*offspring conflict, the resolution of this conflict may shape the duration of critical windows. This perspective may help understand (i) why windows close at different times for different traits, and (ii) why the duration of critical windows may vary across human populations. The issue is explored in relation to population differences in the association between infant weight gain and later body composition. The occupation of more stable environments by western populations may have favoured earlier closure of the critical window during which growth in lean mass is sensitive to nutritional intake. This may paradoxically have elevated the risk of obesity following rapid infant weight gain in such populations.

## INTRODUCTION

Developmental plasticity is well established to play a central role in the aetiology of chronic degenerative diseases, an association now recognized through the ‘developmental origins of health and disease’ (DOHaD) hypothesis [1–5]. In this way, chronic disease risk is understood to be shaped by ecological stresses acting early in development, though adult lifestyle also remains important [6].

The paradox is that despite such implications for long-term disease risk, developmental plasticity is widely considered an adaptive process, often assumed to fine-tune the fit of the organism to its environment. For example, the ‘adaptive calibration’ model assumes that life history strategy is continually shaped by information relating to threats and opportunities during development [7, 8]. A key question therefore is how such an adaptive process can be central to the emergence of ill health.

For chronic diseases, the role of growth patterns in predicting outcome indicates an important developmental role for nutrition in early life. For placental mammals, fetal nutrition is obtained directly from maternal physiology, while postnatal nutrition commences with lactation. As with psychosocial stresses, it is generally accepted that the nutritional supply of the fetus and infant contains ‘ecological information’, and that associations of nutrition and developmental trajectory must in some or other way represent ‘information processing’ [9, 10]. It is more controversial as to what this information comprises, and how the offspring might be able to use it adaptively. In this article, I will focus on growth traits but the same concepts may apply to many other components of development.

The association of developmental plasticity with long-term disease risk can be attributed to the fact that plasticity is of finite duration. Classic studies on rats showed that the effect of undernutrition on growth is different according to when it occurs during development. Exposing rats to undernutrition in early infancy caused a permanent reduction in body size, whereas the same insult later in development only temporarily slowed growth [11, 12]. This work led to the concept of finite ‘critical windows’ of physiological sensitivity, during which many traits, and developmental trajectory in general, are influenced by environmental factors [13–15].

The same scenario applies to humans: for example, by the age of 2 years, height has little sensitivity to ecological conditions, and tends to track within individuals, a process known as canalization [16, 17]. In public health practice, the recognition of finite windows of plasticity has led to emphasis on the first 1000 days from conception [18], which in turn places great importance on information processing during fetal life and infancy. To the extent that the offspring ‘adapts’ its growth trajectory in early life, such adaptation is relatively irreversible.

The importance of the first 1000 days generates a key question: why is the primary period of human developmental plasticity so short, when the total duration of growth (18 years, or ∼6800 days from conception) is so long? Several evolutionary explanations have been offered for the lengthy duration of human maturation, and most of them assume that there are energetic benefits of reducing the rate of growth, thereby distributing its costs over a longer time period. For example, slowed growth has been assumed to allow increased investment in brain growth, and also to reduce the costs of maternal reproduction [19]. Less attention has been directed to the brief duration of critical windows of plasticity, but this also requires an evolutionary explanation.

## PLASTICITY AND INFORMATION PROCESSING

An adaptive perspective considers the offspring to benefit from information by tailoring its life history strategy to local ecological conditions. The ability for life history strategy to respond to ecological signals is well established [20] and is undisputed, but this issue becomes complex once we take into account the origins of these signals in early life.

A crucial issue is that among placental mammals, the fetus does not receive information directly from the external environment, rather it is exposed to maternal phenotype [10]. It is now recognized that maternal physiology is capable of smoothing over short-term signals, such as peaks or troughs in energy availability [10, 21–23]. This smoothing can buffer against ecological stresses such as famine or drought during pregnancy, a process that undeniably benefits the vulnerable offspring, as demonstrated by the restricted falls in birth weight during famines [24, 25].

This maternal smoothing process thus brings the life history strategies of two generations into contact [10]. For over four decades, evolutionary biologists have considered maternal care through the lens of parent–offspring conflict theory, which assumes that nutrients and energy are scarce resources, and that the mother and each offspring are characterized by a conflict of interest regarding how much of her nutritional resources the mother should transfer to the offspring during the period of parental care [26, 27]. This conceptual approach has been applied to human developmental plasticity [10, 23, 28], but further elucidation is merited.

Beyond the physical resources themselves, it should not automatically be assumed that the offspring gains 100% of the pay-offs from the information it receives from its mother [10]. The information that shapes adaptation in growth trajectory can be considered as ‘signals’ transmitted from maternal metabolism to that of the offspring. As discussed by Haig [29], signals that generate no impact on the receiver could be said to contain no information; if they do generate an impact, then that impact derives ultimately from maternal life history strategy. Given such parent–offspring conflict, the transmission of signals through placental nutrition and lactation means that in the process of accessing information, the offspring is also open to maternal manipulation [10, 23].

To date, the majority of attention has been directed to the ‘quantity’ of resources that is transferred during the window of parental care. For example, an early analysis supported the concept of parent–offspring conflict theory, by showing that the most common birth weight is lower than the value that minimizes infant mortality [30]. This suggests that the average mother invests less in birth weight than would be optimal for the offspring. Maternal fitness-maximizing strategy is further evident in investment differences between sons and daughters, a scenario first proposed by Trivers and Willard [31]. In humans, the energy cost of pregnancy is higher for sons than for daughters [32, 33], while studies suggest that either the energy content or volume of milk transferred to male offspring may also be greater than that for females [34, 35]. However, these gender differences require confirmation, and studies of other species show heterogeneity of gender differences in milk energy transfer [36–38], suggesting more than one model of differential investment by gender, or lack of it, should be considered [36].

Beyond the magnitude of investment, maternal strategy is also evident in the ‘timing’ of investment decisions, such as the duration of pregnancy, or the initiation of weaning. These may likewise be considered subject to a conflict of interest: for example, continued gestation or breast-feeding of the current offspring reduces the ability of the mother to invest in the next offspring [39, 40]. Conflict over the timing of life history ‘decisions’ may be addressed at the level of behavior, e.g. conflict over weaning has been explored in the context of infant crying, or more recently night-time suckling [40, 41]. The consequences of how the conflict is resolved can then be considered in physical terms: e.g., the magnitude of growth attained by the offspring, versus the redirection of maternal nutritional reserves to the next offspring [41].

**Figure 1.**
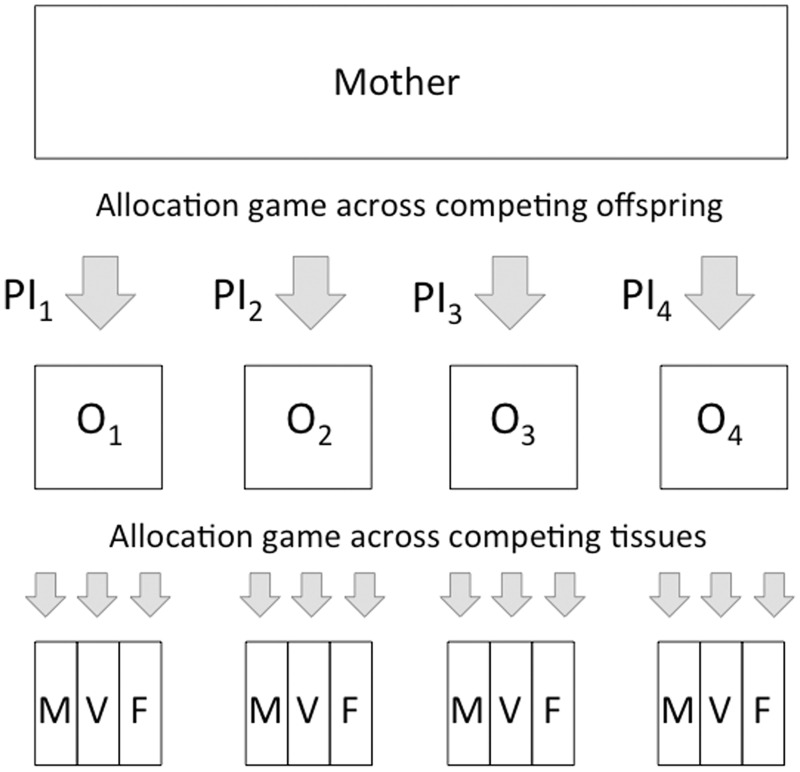
Schematic diagram illustrating two different ‘allocation games’ in which each of the mother and the offspring optimize their inclusive fitness. In the first game, the mother optimizes her allocation of parental investment (PI) across competing offspring (O_1_–O_4_). In the second game, which is sensitive to the first game, each offspring optimizes its allocation of that investment between competing phenotypic traits, such as muscle mass (M), vital organ mass (V) and energy stores in fat (F). These traits also represent allocations across life history functions (maintenance, growth, reproduction and immune function)

Seen through this lens, developmental plasticity cannot be seen simply as a process of the offspring fine-tuning its developmental trajectory to match prevailing ecological conditions; a counter-view is that the mother shapes the growth trajectory of each offspring according to the partitioning of investment that will maximize her own fitness [10]. We can think of two related ‘optimization games’ being solved: the mother must allocate her sum resources for reproduction across multiple offspring, while each offspring must allocate the resources it receives across competing traits (Fig. 1). At a superficial level, these traits may manifest as physical components of growth such as brain size versus energy reserves, but ultimately the offspring is allocating energy between competing life history functions—maintenance, growth, reproductive capacity and immune function. Both mother and offspring gain inclusive fitness benefits from the mother producing multiple offspring, but the optimal point at which the mother ceases to invest in one offspring and begins to invest in another is not equal for mother and offspring [42]. Thus, maternal and offspring strategies are characterized not by outright conflict, but by a ‘conflict of interest’ over the optimal pattern of maternal investment. It is also important to consider the paternal contribution to this conflict: while successive offspring of a given mother are equally genetically related to her, they may be conceived by different fathers. Variability in paternity across a mother’s offspring has the effect of increasing the conflict of interest between paternal genes expressed in the offspring and maternal genes expressed in the mother [43].

Previous work has therefore addressed how parent–offspring conflict characterizes both the magnitude of resources transferred from mother to offspring, and the schedule of this investment. Information transfer occurs within the same context. However, there are also further implications, regarding how long ‘critical windows’ of physiological sensitivity should remain open, and this translates into variability in the ‘source’ of information received by the offspring. To address this issue, let us first consider in more detail how windows of plasticity are linked with maternal care in the first place.

## MATERNAL BUFFERING MATCHES OFFSPRING CRITICAL WINDOWS

I have argued previously that the nesting of windows of plasticity within the period of maternal care is no coincidence [10]. This is because early growth comprises the process of hyperplasia, during which the cells increase in number and thereby shape the structure and long-term function of organs [44]. Environmental factors acting on this period therefore generate long-term effects on vital components of organ phenotype, whereas factors acting later in development affect only the hypertrophic component of growth, and therefore affect size rather than structure.

**Figure 2.**
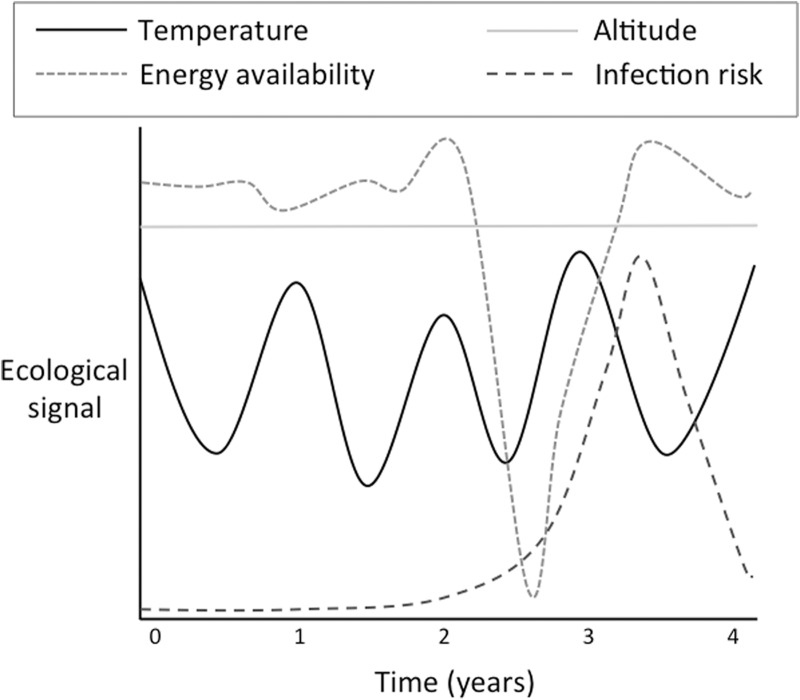
Schematic diagram illustrating contrasting levels of stochasticity in four different ecological parameters. Some parameters such as altitude are relatively stable, others such as temperature may change relatively slowly, whereas others such as infection risk may alter suddenly

It might appear that the offspring would benefit from tailoring its metabolism, tissues and organs directly to prevailing conditions, but such a strategy is extremely challenging because a wide variety of signals is emitted by the environment. These signals include temperature, altitude, local infectious disease burden, energy availability, dietary macronutrient and micronutrient composition, and the psychosocial environment. There may also be transient but powerful stochastic stresses such as floods and droughts (such as those arising from climate cycles such as ‘El Niño’ Southern Oscillation cycles) and volcanic eruptions [45]. Importantly, each of these ecological signals may vary independently in its magnitude and periodicity (Fig. 2). For example, high altitude may represent a relatively consistent stress of hypoxia; temperature may represent a stress with a relatively consistent pattern of seasonal variability; rainfall may be prone to occasional extremes; and energy availability, infectious disease burden and the psychosocial environment may show irregular and random perturbations.

Sampling such a diverse range of signals represents a major challenge for the offspring, as it has not had sufficient time to develop sophisticated physiological sensing mechanisms capable of processing such multiple cues; and yet the period of hyperplasic growth is also the very period when it is most in need of metabolic stability. This dilemma can be resolved by sampling maternal phenotype instead of the external environment, enabling a coherent phenotype to be achieved [10, 22]. Maternal phenotype provides exactly the composite integrated signal of ecological quality that the developing offspring requires. This signal persists through pregnancy, and to a lesser extent through early lactation, when maternal phenotype remains the dominant influence on offspring nutrition, though not the only one (e.g. allo-mothering allows different women to breast-feed a given infant [46, 47]).

Since hyperplasic growth is so sensitive to environmental stresses, we can also assume, in the reverse direction, that maternal care has evolved to buffer the offspring during this period of development. This is achieved through powerful homeostatic physiological mechanisms. The duration of pregnancy is adequate to ensure that the amount of brain growth required for postnatal behavior is completed in utero [48], illustrated by increased risks of impaired cognitive function in offspring born preterm [49], hence the physiologies of maternal pregnancy and fetal development are closely aligned.

Since the mother is directly exposed to diverse ecological signals, what the offspring adapts to is a combination of maternal homeostatic systems that transduce ecological stresses, and elements of maternal phenotype that were shaped during her own development [10, 23, 50]. For example, the effect of hypoxia from high altitude on the developing offspring depends on the nature of maternal accommodation of this stress. A fetus exposed to an ‘adapted mother’ will experience a different *in utero* environment compared to a fetus exposed to an ‘maladapted mother’. The same concept is clearly illustrated by studies of the successive offspring of mothers who at some point develop type 2 diabetes. Offspring gestated after the onset of perturbed maternal metabolism show phenotypic differences to those gestated beforehand, with higher BMI and an elevated risk of themselves developing diabetes [51]. No such elevated risk is evident in relation to the timing of onset of paternal diabetes. Similar findings have emerged from studies of surgical intervention on maternal obesity, where the odds of producing a macrosomic infant decreased postsurgery, while those of producing a small-for gestational age infant increased [52]. Whatever the state of the external environment, it is the ‘mother’s metabolic adaptation to that environment’ that shapes the signals received by the offspring.

What then is the offspring exposed to, and what is it not exposed to? Short-term stresses are transduced and buffered (substantially rather than completely), so that maternal signals are strongly imprinted by ‘maternal capital’, which refers to more stable traits such as uterine volume and energy reserves. Maternal infection impacts the offspring in part by depleting maternal capital [53]. Long-term stresses such as hypoxia or heat/cold stresses are likewise transduced by maternal phenotype.

Many components of maternal metabolism are shaped during the mother’s own development, indicating grand–maternal effects [23, 54]. For example, studies of rodents show that epigenetic effects of maternal diet in the F0 generation may affect not only F1 offspring but also F2 grand–offspring, indicating epigenetic alterations in the germline [55]. In humans, the aggregation of type 2 diabetes in the matriline relative to the patriline is consistent with such a mechanism, although the transmission of mitochondrial DNA would be an alternative possible mechanism [56]. The ooctyes of F1 females are already present in fetal life and are therefore directly exposed to F0 metabolism during pregnancy [57], potentially allowing induced changes in mitochondrial DNA in F1 ooctyes to be transferred through cytoplasm to the F2 generation. In mice, for example maternal insulin resistance has been shown to disrupt oocyte mitochondrial function [58]. Further work is required to elucidate these mechanisms, but it is already clear that offspring accumulate exposure to stable components not only of maternal phenotype, but also longer-term matrilineal influence [10, 22, 23]. It is also becoming clear that through imprinting of the sperm, the offspring is exposed to factors that affected paternal development [59, 60], with paternal food supply during adolescence associated with mortality risk in the offspring [61, 62].

**Figure 3.**
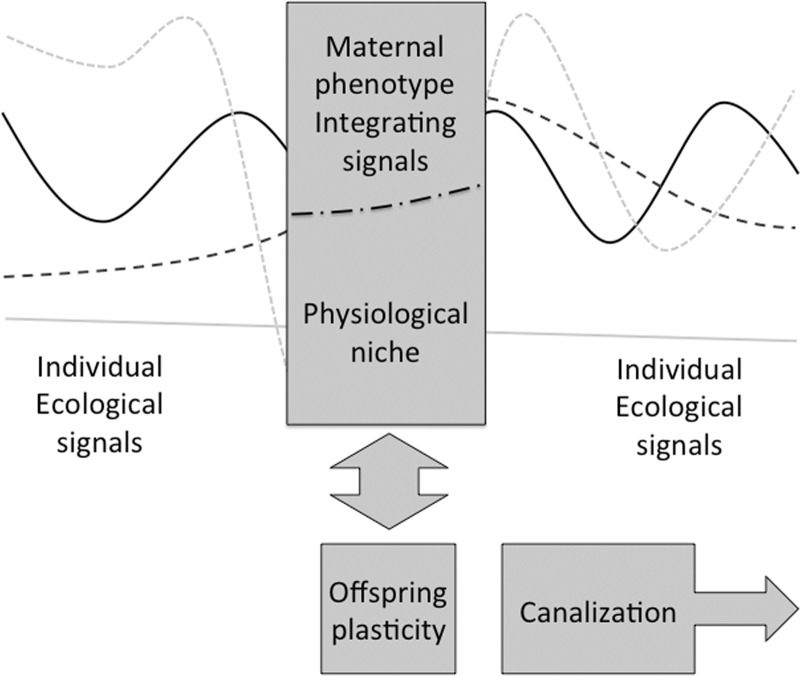
Schematic diagram illustrating how the offspring can resolve the complexity of multiple ecological cues by sampling stable components of maternal metabolism, relying on maternal homeostatic systems to smooth over ecological disruptions. This means that when the offspring is weaned, it must either address all the ecological stochasticity, or develop its own buffering, which it can achieve through closing plasticity and canalizing traits

The exposure of the offspring to maternal phenotype is sharply enhanced at the start of pregnancy, and hence shows sensitivity to maternal diet and nutritional status around the time of conception [63, 64]. Once pregnancy is established, the offspring is exposed to stable components of maternal metabolism. Figure 3 shows how, by responding to signals transmitted by maternal phenotype during pregnancy and lactation, the offspring capitalizes on the mother’s multiple mature homeostatic processes that buffer diverse ecological stresses.

When the mother ceases to provide this stable developmental niche, however, the offspring will be exposed to whatever level of external ecological volatility is present. In order to resist such destabilizing influences, I suggested that the offspring shuts down information processing, and that this accounts for the canalization of many traits around the time of weaning [10].

Support for this model is given by a comparison of traits that are sensitive to maternal physiology during pregnancy and lactation, and those that are sensitive only during pregnancy. For example, offspring hemodynamics are in direct contact with maternal hemodynamics during the period of placental nutrition, however this link is lost at the time of birth. This may explain why the number of nephrons, related to blood pressure regulation, is ‘fixed’ at birth, and cannot change in response to nutritional signals in postnatal life [65]. In contrast, the offspring remains exposed to cues of energy availability through lactation, and the period during which nutritional cues shape development of the pancreas therefore extends into infancy [66, 67]. Infants exposed to poor maternal homeostasis, for example mothers with diabetes, develop long-term metabolic perturbations. This has been attributed to excess levels of sugar in the breast milk [68, 69].

This perspective provides a theoretical explanation for the variable duration of critical windows in physiology: they should persist during the umbrella of maternal care, and then close as this care is withdrawn [10]. A key question therefore is whether different levels of maternal care might be favoured in different ecological settings, and if so, whether this might further affect the duration of offspring plasticity. For example, in systematically poorer-quality environments mothers can be assumed to have fewer resources available for investment, necessitating increased thriftiness in the offspring [23]. This is demonstrated by shifts in the ratio of fat to lean tissue in low-birth weight Indian neonates [70]. However, environments also vary in their stability as well as their quality, so a related issue is whether variability in the stability of ecological conditions should impact the duration of critical windows.

Both mother and offspring share an interest in protecting the offspring during its most vulnerable stage of development, to ensure it develops a viable phenotype. On the basis of maximizing inclusive fitness [71, 72], both parties also benefit from the mother eventually switching investment to the next offspring. Three key points can now be made regarding this switch.

- Since the offspring must canalize its phenotype when maternal investment ceases, the maternal strategy for making this switch should determine the duration of the critical window.
- The optimal timing of this switch is predicted to vary between the two parties, and parent–offspring conflict during infancy is expected to be stronger than during pregnancy, as lactation is more costly to the mother in energetic terms than gestation [73].
- The quality of the environment may affect the optimal schedule (for both mother and offspring, but potentially in different ways) regarding the switching of investment to the next offspring.

Bearing in mind these three points, we can now reconsider the conflict of interest between mother and offspring over the duration of maternal care, and its impact on offspring plasticity. Haig previously developed a model of the optimal duration of pregnancy, illustrating how the offspring favours a longer gestation than the mother, because, sharing only 50% of its genes with its mother, it discounts some of the fitness costs that are born by the mother if she extends the current pregnancy [39]. Haig [39] also noted that the shape of the fitness function would potentially influence the magnitude of the conflict of interest.

**Figure 4.**
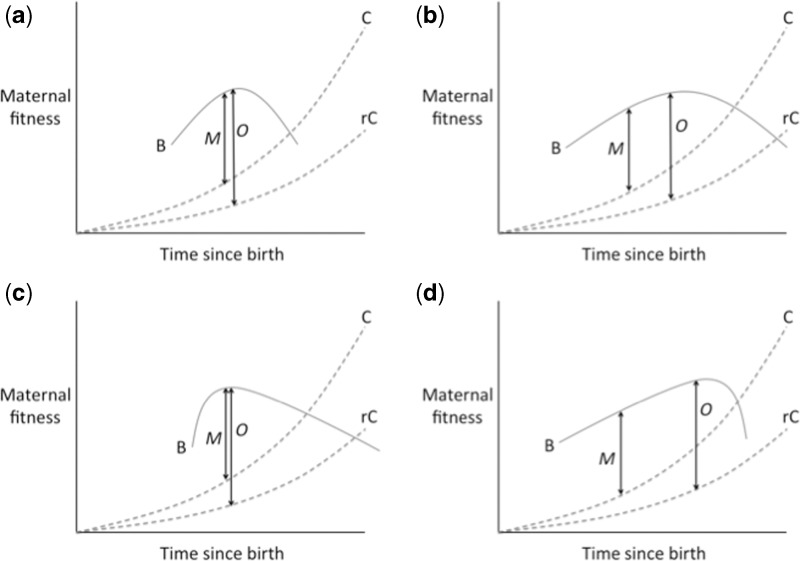
Schematic diagram illustrating how the mother and the offspring have different optima for the duration of maternal care, according to the maternal fitness function (B), based on Haig’s model of optimal pregnancy duration [39]. The cost of continuing investment in the current offspring is given by C for the mother, but by rC for the offspring, which discounts some of these costs as it shares only 50% of its genes with its mother. Maternal fitness (*M*) is maximized by B–C, while offspring fitness (*O*) is maximized by B–rC. According to ecological conditions, the entire fitness function B may be shifted earlier or later, or the shape of the function may change, as shown in scenarios (a)–(d) and discussed in the article text

We can apply this approach to the optimal duration of maternal care during infancy. Figure 4 shows four different fitness functions, and their influence on the magnitude of the conflict of interest. If the fitness function has a sharp peak (Fig. 4a), then this forces the maternal and offspring optima to converge, thereby reducing the conflict of interest. Flattening the peak (Fig. 4b) allows the optimum of the offspring to diverge from that of the mother; this then predicts an increased duration of conflict between the two parties.

However, these scenarios assume that the peak is relatively symmetrical, whereas it is also possible for the peak to show a skewed shape. In Fig. 4c, the peak is right-skewed, indicating that it is very costly to maternal fitness if investment is withdrawn too early, but not if it is withdrawn much later. This scenario may occur when the offspring is very dependent on a maternal resource, or on buffering, up to a certain stage of development. In Fig. 4d, the reverse is shown. This scenario may occur if maternal investment or protection suddenly becomes of little value to the offspring, so that the benefits of switching to the next offspring rise rapidly.

This conceptual approach highlights how ecological conditions may affect the optimal duration of maternal care for each of the two parties, and hence influence the conflict of interest between them. Resolving this conflict in accordance with local ecological conditions may then shift the duration of the critical window backwards or forwards in terms of the stage of offspring development. What evidence is there that such variable duration of critical windows does exist?

## EVOLUTIONARY TRENDS

At the broadest level, evolutionary trends in maternal care may have reshaped the profile of human developmental plasticity. While the total duration of growth lengthened in comparison with other apes during human evolution, the duration of pregnancy is very similar across these species, while the duration of lactation may have shortened in response to several stresses (Table 1) [48, 74]. The duration of critical windows may not map neatly onto the complete period before weaning, but these data do indicate a compression of the duration of maternal care in humans relative to other apes, complementary to the overall elongation of development.

**Table 1.** Contributions of the components of maternal care (gestation and lactation) to total duration of development in apes^a^

Species	Gestation (years)	Weaning age (years)	Total period of care (years)	Age at first birth (years)	Gestation/ development (%)	Total care/ development (%)
Orangutan	0.71	7.0	7.7	15.6	4.4	47.3
Gorilla	0.70	2.8	3.5	10	6.5	32.7
Chimpanzee	0.62	4.5	5.1	13.3	4.5	38.6
Bonobo	0.65	4.5	5.1	13	4.8	37.7
Humans	0.73	2.8	3.5	19.5	3.6	17.4

^a^Duration of development taken as the sum of age at first birth and gestation. Total period of care taken as the sum of gestation and weaning age.

Several explanations have been proposed for reductions in the duration of lactation in the genus *Homo*, including the evolution of larger body size, and the emergence of cooperative breeding, each of which altered the fundamental energetics of reproduction [75, 76]. Within more recent human evolution, the emergence of agriculture may have further reduced lactation and shortened the average birth interval [77–80]. As discussed below, this issue could potentially be investigated by studying contemporary human populations with varying patterns of maternal care. Before considering this, let us consider what aspects of physiology might show such effects.

## VARIABLE CRITICAL WINDOWS

One candidate for the variable duration of critical windows may be the tissues targeted by growth and nutrition during early infancy. Studies in multiple populations have shown relatively consistently that birth weight is strongly associated with later height and lean mass, whereas associations of birth weight with later adiposity are inconsistent, and appear dependent on patterns of postnatal growth [81]. The implications of this research are that the primary long-term target of fetal growth is lean mass, though there is also some indication that this is more the case for males than females [82–84]. This indicates that the two sexes have different optimal investment strategies, reflecting contrasting associations of fat and lean tissue with fitness in adulthood [85, 86].

In infancy, however, studies have shown contrasting results from industrialized populations and those from developing countries [81]. In industrialized populations, faster infant weight gain has been associated with later adiposity, and hence with obesity risk [87–93], whereas in developing countries, infant weight gain shows little or no association with later adiposity, and instead is associated with later lean mass [83, 84, 94–96]. In Brazilian children, for example an association between weight gain and subsequent fat mass was not evident in relation to birth weight or infant weight gain from 0 to 6 or 6 to 12 months, and only emerged from 1 year of age [94].

These contrasting findings suggest that the duration of plasticity during which lean mass is sensitive to cues of energy supply may vary across populations. Human growth has been characterized using an ‘Infancy, Childhood, Puberty’ model, which considers that there are successive stages, that do not however have clearly demarked starts and ends, but rather merge one with the next [97]. During the infant stage, growth has high sensitivity to the hormone insulin, and hence to nutritional intakes, whereas during the childhood period, this nutritional influence declines and growth becomes regulated by the growth hormone (GH)—IGF1 axis, which effectively canalizes growth. Variability in the transition between the infant and childhood stages might either reflect selection of different genotypes, or it might reflect regulation of hormonal axes on the basis of cues during development [98, 99]. Either way, ecological conditions would thereby shape the transition from one stage of growth to the next, and hence shape the schedule over which growth becomes canalized. In other words, this would introduce population variability into the duration of critical windows during which growth patterns are sensitive to nutrition.

Such a mechanism could potentially explain the population variability in the allocation of energy to lean versus fat tissue during early life, and long-term associations between early growth and later body composition. Hochberg and Albertsson-Wikland [98] speculated that a delayed transition to the childhood period of growth would be favoured by ecological stresses such as infectious disease and energy scarcity. While in poor environments this may render infants from such populations prone to long-term stunting, as reduced growth during infancy as well as fetal life would permanently reduce adult height, the counterpart is that faster weight gain during these periods would also benefit height and lean mass, as the data described above have shown. In contrast, infants from industrialized populations may have evolved an earlier transition to GH-regulated growth, as the overall lower burden of infectious disease may have favoured earlier weaning decisions by mothers, and hence earlier closure of the critical window comprising the infant period of growth. Such a strategy would then prevent increased infant energy supply from being directed to greater height and lean mass, and would instead promote excess fat accumulation. In other words, reshaping of the ‘programming of lean mass’ may exert influence on the ‘programming of adiposity’. In industrialized populations, increases in energy supply during childhood are associated with faster growth and greater weight and fatness, but not with greater final size [100].

## OPPORTUNITIES TO TEST THE HYPOTHESIS

Experiments in non-human animals represent one opportunity for testing the hypothesis outlined above. Studies on cognitive development have already noted links between the schedule of brain development and the pattern of maternal care. For example, behavioral imprinting in birds and mammals can only occur after hatching or birth, respectively [101]. However, species might differ substantially as to whether adaptation in critical windows occurred through genetic versus non-genetic mechanisms, making it difficult to interpret results in the context of human physiology.

Amongst humans, several axes of behavioral variability are relevant. Contemporary foragers maintain substantially longer duration of lactation than farmers, though they may also have different infectious disease burdens. Amongst the Bofi of the Central African Republic, for example foraging populations wean their offspring at 36–53 months, whereas their farmer neighbours wean substantially earlier at 18–27 months [102]. Such behavioral differences might be associated with variable durations of critical windows.

A more widespread source of variability is infant feeding mode, where differences in breast-feeding duration, feeding style and milk composition might each generate contrasting signals received by the offspring. Breast-fed and formula-fed infants have been shown to differ in their accretion of fat and lean tissue in early life [103, 104], and to have different risk of obesity in the long term [105–107]. In addition, associations between breast-feeding and later obesity risk are heterogeneous across populations [108], which may relate to inconsistency between settings in either breast-feeding or formula-feeding practices. The possibility that infant feeding mode shapes the duration of critical windows is therefore another area meriting attention.

## PUBLIC HEALTH IMPLICATIONS

As the duration of growth extended during the evolution of *Homo* and humans, the duration of maternal physiological care (placental nutrition and lactation) appears to have remained relatively brief, and to have shortened further since the origins of agriculture. If the arguments presented above are correct, this early cessation of maternal buffering is likely to have constrained the duration of early windows of plasticity, a key period of epigenetic adaptation. This would help explain why this brief period of development is so important for long-term health and function, as is increasingly demonstrated by epidemiological and trial evidence [4]. The short duration of early critical windows does not preclude phenotype responding to ecological stresses during subsequent periods such as adolescence, but the physiological effects are likely to be different to those that occur during early hyperplasic growth.

The notion that the ‘programming’ of obesity need not necessarily be constant across populations has important implications for public health policies. An evolutionary perspective emphasizes that biology may vary in relation to a variety of adaptive processes in response to local ecological conditions, and to intrinsic factors such as gender and age. Traditionally, metabolic differences between populations were explained using the ‘thrifty genotype’ hypothesis, which assumed that ancestral populations had varied in their exposure to ‘feast-famine’ cycles, and had evolved contrasting genotypes [109]. More recently, the thrifty phenotype hypothesis emphasized the role developmental experience in shaping subsequent metabolism, independent of genotype [6]. The mechanism described above may involve either genetic adaptation, or mechanisms of plasticity, and at this stage neither can be ruled out.

This perspective therefore highlights important gaps in knowledge regarding public health policies that target stunting and obesity risk in early life:

- If population variability in the duration of critical windows were attributable to genetic factors, then different policies would be required for different populations.
- If the variability emerged through plasticity, whereby different patterns of maternal care shape the developmental programming of hormonal axes, then a public health intervention might impact not only nutritional status *per se*, but also the fundamental physiology of growth.

The possibility that public health policies might impact the very regulatory systems that underlie the traits that they are seeking to influence is clearly challenging, but this is precisely the kind of insight that an evolutionary perspective using life history theory can contribute to mainstream medicine.

## CONCLUSIONS

In this article, I have developed earlier work on the notion that developmental plasticity has been selected to match with the duration of maternal care by considering the additional influence of ecological conditions [10]. I have suggested that variability in ecological quality, represented by stresses such as the burden of infectious disease and the stability of energy supply, could shape patterns of maternal investment, and that this could then elicit population variability in the duration of critical windows of physiological sensitivity. In this model of dynamic interactions between mother and offspring, neither party should be assumed to have total control. Mothers have some ability to alter their patterns of parental care to benefit their own inclusive fitness, but only to the extent that their decisions do not fatally compromise offspring viability and survival. As elegantly expressed in the concept of the tug-of-war over maternal investment [27], mothers may shift their reproductive strategy if the returns on investment in the current offspring increase or decline in accordance with ecological conditions, and in this way may affect the duration of critical windows. This issue requires further work, but is important as it may have implications not only for what public health policies might achieve in terms of improving growth and nutritional status, but how they might influence more fundamental components of human biology in the process. This issue is relevant to the key challenge of reducing stunting and malnutrition without exacerbating longer term risks of obesity.

**Conflict of interest**: None declared.
